# Harnessing Big Heterogeneous Data to Evaluate the Potential Impact of HIV Responses Among Key Populations in Sub-Saharan Africa: Protocol for the Boloka Data Repository Initiative

**DOI:** 10.2196/63583

**Published:** 2025-01-22

**Authors:** Refilwe Nancy Phaswana Mafuya, Edith Phalane, Amrita Rao, Kalai Willis, Katherine Rucinski, K Alida Voet, Amal Abdulrahman, Claris Siyamayambo, Betty Sebati, Mohlago Seloka, Musa Jaiteh, Lerato Lucia Olifant, Katharine Journeay, Haley Sisel, Xiaoming Li, Bankole Olatosi, Neset Hikmet, Prashant Duhoon, Francois Wolmarans, Yegnanew A Shiferaw, Lifutso Motsieloa, Mashudu Rampilo, Stefan Baral

**Affiliations:** 1 South African Medical Research Council/University of Johannesburg Pan African Centre for Epidemics Research Extramural Unit University of Johannesburg Johannesburg South Africa; 2 Department of Health Services Policy Management Arnold School of Public Health University of South Carolina Columbia, SC United States; 3 Key Populations Program, Center for Public Health and Human Rights, Johns Hopkins Bloomberg School of Public Health Johns Hopkins University Baltimore, MD United States; 4 Big Data Health Science Center, Arnold School of Public Health University of South Carolina Columbia, SC United States; 5 Engineering and Computing, Integrated Information Technology University of South Carolina Columbia, SC United States; 6 Technology Architecture & Planning University of Johannesburg Johannesburg South Africa; 7 Department of Statistics, Faculty of Science University of Johannesburg Johannesburg South Africa; 8 South African National AIDS Council Pretoria South Africa

**Keywords:** HIV, key populations, Sub-Saharan Africa, big heterogeneous data, Boloka data repository

## Abstract

**Background:**

In South Africa, there is no centralized HIV surveillance system where key populations (KPs) data, including gay men and other men who have sex with men, female sex workers, transgender persons, people who use drugs, and incarcerated persons, are stored in South Africa despite being on higher risk of HIV acquisition and transmission than the general population. Data on KPs are being collected on a smaller scale by numerous stakeholders and managed in silos. There exists an opportunity to harness a variety of data, such as empirical, contextual, observational, and programmatic data, for evaluating the potential impact of HIV responses among KPs in South Africa.

**Objective:**

This study aimed to leverage and harness big heterogeneous data on HIV among KPs and harmonize and analyze it to inform a targeted HIV response for greater impact in Sub-Saharan Africa.

**Methods:**

The Boloka data repository initiative has 5 stages. There will be engagement of a wide range of stakeholders to facilitate the acquisition of data (stage 1). Through these engagements, different data types will be collated (stage 2). The data will be filtered and screened to enable high-quality analyses (stage 3). The collated data will be stored in the Boloka data repository (stage 4). The Boloka data repository will be made accessible to stakeholders and authorized users (stage 5).

**Results:**

The protocol was funded by the South African Medical Research Council following external peer reviews (December 2022). The study received initial ethics approval (May 2022), renewal (June 2023), and amendment (July 2024) from the University of Johannesburg (UJ) Research Ethics Committee. The research team has been recruited, onboarded, and received non–web-based internet ethics training (January 2023). A list of current and potential data partners has been compiled (January 2023 to date). Data sharing or user agreements have been signed with several data partners (August 2023 to date). Survey and routine data have been and are being secured (January 5, 2023). In (September 2024) we received Ghana Men Study data. The data transfer agreement between the Pan African Centre for Epidemics Research and the Perinatal HIV Research Unit was finalized (October 2024), and we are anticipating receiving data by (December 2024). In total, 7 abstracts are underway, with 1 abstract completed the analysis and expected to submit the full article to the peer-reviewed journal in early January 2024. As of March 2025, we expect to submit the remaining 6 full articles.

**Conclusions:**

A truly “complete” data infrastructure that systematically and rigorously integrates diverse data for KPs will not only improve our understanding of local epidemics but will also improve HIV interventions and policies. Furthermore, it will inform future research directions and become an incredible institutional mechanism for epidemiological and public health training in South Africa and Sub-Saharan Africa.

**International Registered Report Identifier (IRRID):**

DERR1-10.2196/63583

## Introduction

In 2024, the world is at a critical juncture in the HIV response, counting down 6 years toward the global goal of ending AIDS as a public health threat by 2030 [1,2]. Despite the investment and focus on addressing HIV/AIDS, it remains a significant public health threat with persistent prevention and treatment challenges globally, regionally, in Sub-Saharan Africa, and in South Africa [1-3]. South Africa has the largest HIV epidemic in the world, with about 8 million people living with HIV, which represents approximately 1 in 5 of the estimated 38.4 million people living with HIV globally in 2022 [1,4]. While there has been a steady decline of 30.5% (n=198,311) in new infections in the last 5 years, the country still has an unacceptably high HIV incidence (South African National HIV, Prevalence, Incidence, Behaviour, and Communication Survey) [3-5]. South Africa has the largest HIV treatment program in the world to meet the treatment needs of the highest proportion of people living with HIV [1].

Key populations (KPs), including female sex workers and their clients, gay men and other men who have sex with men, transgender people, people who use and inject drugs, and incarcerated persons, face a disproportionate risk of HIV acquisition and onward transmission [6]. As a result of unmet prevention and treatment needs, 51% of new HIV infections are acquired by key populations and their sexual partners despite making up approximately 1.5% of the total adult population in Sub-Saharan Africa [1]. The estimated prevalence of HIV was 59.5% among female sex workers and 29.7% among men who have sex with men in South Africa in 2020 [3,4]. Given social network dynamics, the overall impact of the unmet needs of key populations on onward transmission may be even greater. Other modeling studies have a higher risk of onward transmission due to the unmet prevention and treatment needs among key populations using the transmission population attributable fraction over time (tPAF_t_) [7]. This demonstrates the need for the specificity of the HIV response in characterizing and addressing heterogeneity in onward transmission for a more significant impact in the reduction of new infections. This disproportionate risk of HIV is driven by discrimination, stigma, and criminalization of behavior [8,9]. These same factors, alongside social network dynamics, make collecting data on HIV burden and specific HIV prevention and treatment needs challenging due to distrust of institutions and fear of poor treatment, arrest, or violence [10-12].

In South Africa, where there is robust HIV surveillance compared with other Sub-Saharan African countries, there is no specific mechanism or centralized system to gather and monitor key population data. The existing HIV surveillance systems include the district health information system (DHIS) and the integrated electronic registers, which both collect patient-level HIV data but have no identifiers for KPs, making it difficult to disaggregate data by these subpopulations [13]. The inability to disaggregate information among KPs can lead to misallocation of resources and services, perpetuating health inequities. This lack of adequate data can also lead to underestimation of the disproportionate risk of onward transmission and ultimately missed opportunities for targeted approaches that can lead to a significant reduction of new HIV cases [7,14,15]. Despite this lack of a centralized system, data on KPs are being collected on a smaller scale by numerous stakeholders, including program implementers, the government, academic partners, and others. There exists an opportunity to harness these different data sources for integration and in-depth analyses.

In generalized epidemic settings, there has been a tendency to focus the HIV response on the general population rather than KPs [16-18]. The scientific justification for the project presented here is that continued reliance on nonspecific population-based approaches to guide programs has limited the broader impact of the HIV response in a generalized epidemic setting like South Africa. At the same time, there is limited data to determine the extent to which a KP-tailored HIV response could reduce HIV incidence in South Africa [6,7,19-21]. A more specific HIV response will likely optimize the use of limited resources [7,20]. The proposed work can support program implementers, funders, and policy makers in making well-informed choices as to where, what, and whom to prioritize and how to deliver effective HIV control programs to maximize health benefits at a population level. An effective control of the HIV epidemic in South Africa requires a focus on KPs [22].

To address the identified gaps in the field, we are in the process of designing and developing a big data platform called “The Boloka data repository” and harmonization of disparate data from multiple data sources. The Boloka data repository seeks to store a diverse range of data, including empirical, observational, and programmatic data, to improve understanding of HIV acquisition and transmission among KPs. In addition, the Boloka data repository seeks to evaluate the potential impact of HIV responses in South Africa in the context of a generalized epidemic setting. Boloka is a South African Sepedi, Sesotho, and Setswana word that means to “store or keep*.*” In this case, we will store or keep big heterogeneous data. These data, including HIV-related and relevant data for KPs from the year 2000 onward in South Africa, can be used to inform policy and programming. By harnessing big heterogeneous data, more data-driven and ultimately more effective HIV response strategies can be generated.

In this protocol paper, we describe the process for developing the Boloka data repository for South Africa. It is envisaged that once the Boloka data repository has been developed, it can be adapted to other countries throughout Sub-Saharan Africa. This study has 3 specific objectives: First, to build a Boloka data repository to handle data on KPs. Second, to collate available HIV-related data for KPs in South Africa from 2000 onwards. Third, to make the data user-friendly for use by stakeholders and authorized users.

## Methods

### Overview

**Figure 1:**
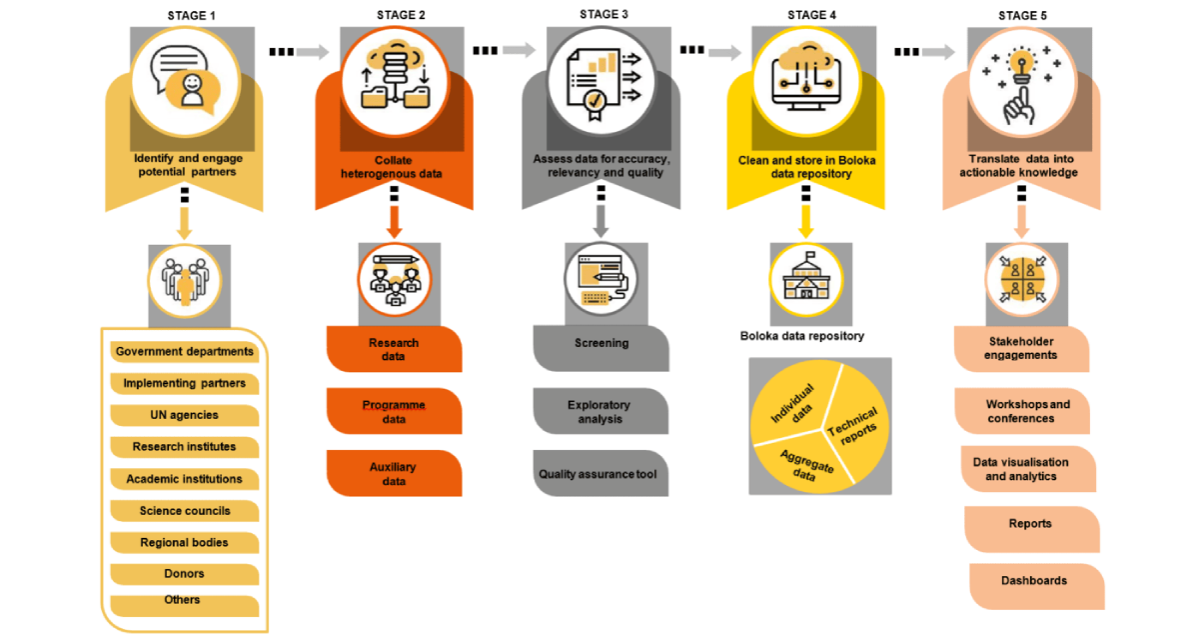
The 5 stages of the Boloka data repository initiative [23], with permission from RNPM. UN: United Nations.

To achieve the stated objectives, this study project will be developed in the following 5 key stages (Figure 1) [23].

### Stage 1: Engage Key Stakeholders to Develop Meaningful Data Partnerships

A range of stakeholders (potential data partners) will be engaged to explore data sharing partnerships and facilitate the collation as well as the organization of the diverse data sources (Table 1). A list of current and potential data sharing partners and the nature of data they are in possession of is being developed (Multimedia Appendix 1 shows the list of potential and current data partners as well nature of data).

A key stakeholder in this work is the South African National AIDS Council (SANAC), which will act as a partner in both the sharing of data and the facilitation of connections to additional potential data sharing partners. A transdisciplinary participatory approach will be adapted to develop meaningful partnerships ensuring collaboration between researchers and data partners, buy-in, project performance, co-ownership, and sustainability [24-26] guided by key principles of mutual trust, sharing, transparency, and responsibility [25,26]. Stakeholders will be given an opportunity to give input at all stages about various components of the Boloka data repository, including which data are collected, how they are stored, data confidentiality, data privacy, and data security, as well as how others may access them using the approved data management processes of the respective data partners. The Boloka research team will also engage with stakeholders regarding priority research questions that will be answered using the data (January 2024 to December 2026). This will ensure that the questions asked have use for those directly involved in the HIV response.

The data processing agreement (DPA) and data partnership or user and related agreements will be signed between the University of Johannesburg and the respective data partners [27]. These documents will govern the rights and duties of the parties in line “Protection of Personal Information (POPI) Act number 4 of 2014,” henceforth referred to as the POPI Act, and any other applicable data protection, security, storage, regulation, and legal requirements [28]. Through these agreements, it will be ensured that the collection, storage, use, disclosure, transfer, disposal, and other processing of any personal information is in line with the prescribed data protection law. The signed agreements will be stored at the University of Johannesburg records management unit.

The Boloka data repository initiative will be executed in the South African Medical Research Council/ UJ (SAMRC/UJ) Pan African Centre for Epidemics Research (PACER) Extramural Unit, which is part of over 40 research centers and institutes, and 20 prestigious national research and industry-funded chairs that the University of Johannesburg is hosting. The South African Medical Research Council/ UJ (SAMRC/UJ) Pan African Centre for Epidemics Research Extramural Unit is one of the flagship initiatives that is supported under the Global Excellence and Stature strategic initiative [29].

**Table 1:** List of current and potential Boloka Project data partners.

Partner category	Examples of data partners
Country coordinating mechanism	SANAC (South African National AIDS Council)
International and Regional UN Agencies	Centers for Disease Control and Prevention (CDC), UNICEF, United Nations Development Programme, UNAIDS, WHO, United Nations Population Fund.
Government Departments	National Departments of Health, Social Development, Correctional Services, Basic Education.
Nongovernmental organizations (NGOs), nonprofit organizations (NPOs), and other	Beyond Zero (BZ), Networking HIV & AIDS Community of Southern Africa (NACOSA), Sex Workers Education and Advocacy Taskforce (SWEAT), Aurum, TB/HIV Care, Right to Care, Sisonke, U.S. President’s Emergency Plan for AIDS Relief (PEPFAR) funded organizations.
Science Councils	Human Sciences Research Council (HSRC), South African Medical Research Council, Council for Scientific and Industrial Research.
Research Institutes in Universities	Desmond Tutu HIV Foundation, Centre for the AIDS Programme of Research in South Africa, Perinatal Health Research Unit.
Local and international donors	Global Fund, President's Emergency Plan for AIDS Relief (PEPFAR), UNAIDS, European Union (EU), Department for International Development (DFID), Canadian International Development Agency (CIDA), Directorate-General for International Cooperation (DGIS), Bill and Melinda Gates Foundation (BMG).
National Universities	All South African Universities.
Regional economic communities	Southern Africa Development Community.

### Stage 2: Acquire and Collate Heterogeneous Data

#### Overview

The Boloka data repository will leverage and collate available and diverse data from various sources across places, times, and populations (Multimedia Appendix 1 shows the list of potential or current data partners and data types). The inclusion and exclusion criteria (Textbox 1), as well as the Boloka data indicator tool (Multimedia Appendix 2), will guide the eligibility of the data source. The inclusion of HIV-related data from the period 2000 and onwards will enable the capturing of the developments that took place over time in the HIV field. The use of reliable and granular data collected over time is essential for improving population health, setting targets as well as developing strategies for targeted HIV response.

**Figure 2:**
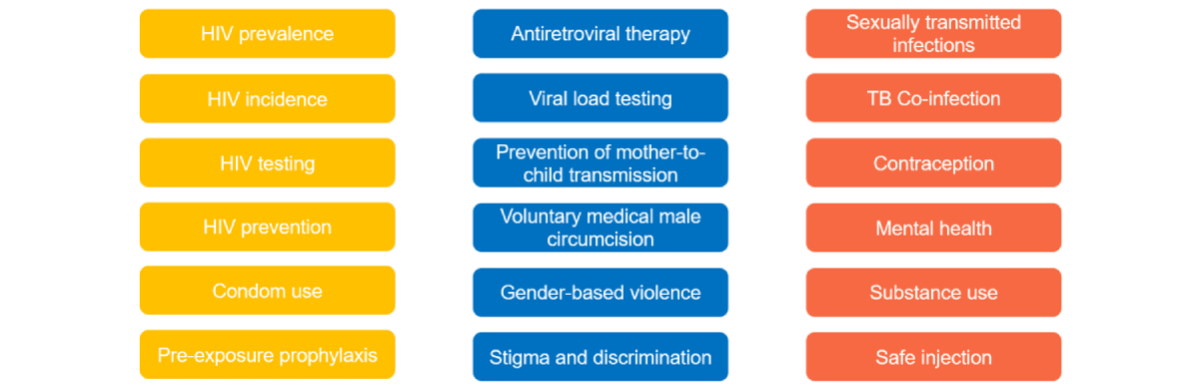
Boloka HIV-related indicator list.

The team reviewed the Joint United Nations Programme on HIV/AIDS global monitoring tool and the National Department of Health (NDoH) national indicator dataset form used for global, regional, and national HIV reporting to identify primary and secondary indicators in line with global, regional, and national priorities. The team also reviewed existing validated questionnaires or instruments from various data partners to further refine priority indicators; from this, broader categories of HIV-related interest areas were formed (Figure 2). Accordingly, each potential data partner is required to indicate the primary and secondary indicators that they have collected data on the Boloka data indicator tool (Multimedia Appendix 2).

Where possible, data will be disaggregated at individual, facility, district, or sub-district levels to enable advanced analyses that are not possible with higher-order aggregated data. This will help us develop an in-depth understanding of various subsets of the populations within the larger datasets. Where data are aggregated, we will seek data disaggregated by sex, gender, age groups, socioeconomic status, geo-location, facility type, and temporal factors to understand a range of HIV indicators, including heterogeneity of HIV risk and burden, engagement in HIV services (treatment cascade, pre-exposure prophylaxis uptake and continuation), and population size estimates, among others. The process for requesting and accessing data varies based on the data type and institution. As such, the process of acquiring and collating the data for the different data types is discussed below.

**Inclusion and exclusion criteria.**

Inclusion criteria:

- Study topic: HIV, key populations, HIV prevention and treatment.
- Study area: South Africa.
- Language: English.
- Time frame: Collected in the year 2000 or later.
- Data type: Research and program data.
- Data regulation: Secondary dataset or study with primary consent, specifying that the original consent or ethics approval covers secondary analysis without additional consent from participants.

Exclusion criteria:

- Study topic: Non-HIV.
- Study area: Non–South Africa.
- Language: Non-English.
- Time frame: Collected before the year 2000.
- Data type: Social media data.
- Data regulation: In circumstances where primary consent is needed from the participants, such datasets or studies will be excluded as it may not be feasible to go back to the participants for reconsent.

#### Research Data

For open access data, which is typically aggregated or deidentified individual data, we will adhere to procedures set by the respective institutions to obtain access in line with the POPI Act. This may require the submission of designated data access request forms before gaining direct access to the dataset. For data that are only available upon request, we will contact the data partner to understand their specific procedures for accessing data. This typically requires the completion of a data access request form and the sharing of relevant information, such as the project proposal, ethics approval, and timelines for analysis. For institutions such as the NDoH, district health information data is made available for public use upon request and completion of their data user agreement (DUA) forms. The data or indicators requested should be aligned with the approved National indicator dataset, which outlines the data elements and indicators collected by the NDoH. In such cases, the request and DUA forms will be completed to access data. For institutions in possession of data that are not typically available for public access (eg, implementing partners), we will seek to develop formal partnership agreements and additionally use the University of Johannesburg DPA to ensure adherence to the Protection of Personal Information Act in terms of data privacy and security before any data sharing. Once agreed upon, a formalized partnership agreement will be approved by the legal team of both parties. These agreements will be reviewed in accordance with the policies of the respective organizations.

#### Program Data

For institutions in possession of data that is not typically available for public access, we will seek to develop formal partnership agreements and additionally use the University of Johannesburg DPA to ensure adherence to the POPI Act in terms of data privacy and security before any data sharing. Once agreed upon, a formalized partnership agreement approved by the legal team of both parties will be signed. These agreements will be reviewed in accordance with the policies of the respective organizations.

### Stage 3: Screen Data for Relevance and Quality

#### Overview

Once data are acquired and determined to be eligible according to the inclusion and exclusion criteria (Textbox 1), they will go through a process of screening and assessment for accuracy, relevance, quality, completeness, and consistency before inclusion in the Boloka data repository. Due to limited information and validated tools on how to specifically check for relevance and quality for some of the data types [30], we will adopt the Framework for Data Quality developed by the Federal Committee on Statistical Methodology (FCSM) [31] and the Information Quality Assessment Framework [32]. Below are further details on the data screening process by the different data types.

#### Research Data

For research data, a multistep quality assessment process will be carried out using the Global HIV Quality Assessment Tool (Multimedia Appendix 3) [33]. This tool will be used to review and verify the suitability and quality of research data sources in terms of study design and implementation, along with criteria for HIV indicators, specifically prevalence, incidence, engagement in the HIV care continuum, and population size estimates [33]. There will be close supervision and checks to minimize errors by using an honest data broker mechanism to manage and maintain datasets. In addition, 2 individuals (research assistants) will perform the initial quality assessment. Any discrepancies identified will be addressed by a third assessor (Project Manager and Principal Investigator [PI]). Exploratory analyses will be conducted to understand the nature of the data and identify outliers and missing data that will be cleaned for data quality and accuracy. This will be an iterative process to ensure that we leverage and assemble the best available data.

#### Program Data

It is important to highlight that program data present unique data quality challenges. Program data are typically aggregated to administrative units, and information on individuals is generally not available. Hence, it is not always feasible to link program exposure directly to an outcome [34]. In terms of screening the program data for inclusion into the Boloka data repository, the World Health Organization’s “Data quality review: a toolkit for facility data quality assessment—Module 1: Framework and metrics” will be adapted for use [35]. This framework is made up of 4 dimensions. For this study, dimensions 1 and 2 will be used: completeness and timeliness of the data (dimension 1) and internal consistency of reported data (dimension 2). Dimension 1 focuses on the data elements and indicators collected. For example, the NDoH and implementing partners’ data elements and indicators will be assessed and checked for completeness [35]. Dimension 2, that is, the internal consistency of the data, relates to the coherence of the evaluated data. In this regard, 4 metrics of internal consistency will be used [35]: (1) presence of outliers, (2) consistency over time, (3) consistency between indicators, and (4) consistency of reported data.

In this initial screening, articles, reports, and datasets will be excluded if their scope does not adhere to the inclusion criteria mentioned in Textbox 1. Data that focuses on the general population will be included in addition to data with a focus on KPs, as the general population provides important KP contextualization. Furthermore, data that have been empirically collected will be prioritized. Estimates and modeled data will be included but carefully reviewed to ensure they meet quality standards and are deduplicated within the datasets.

A report detailing the results of the quality assessment will be written. The report will include the recommendations for data quality improvement plans considering the context and existing constraints. A feedback loop will be used to engage with stakeholders on the potential data quality issues and anomalies, such as completeness of data, discrepancies, and inaccuracies. In addition, the data quality results will be available and stored alongside the data in the repository as part of the metadata.

### Stage 4: Clean and Store Data In A Data Repository

#### Overview

**Figure 3:**
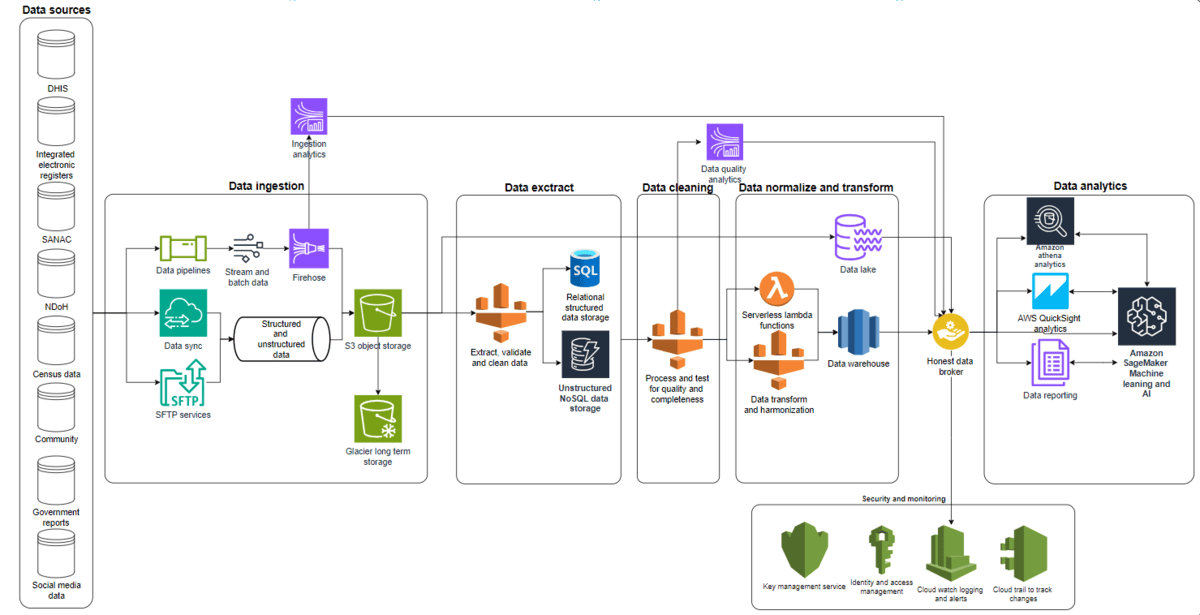
Envisaged Boloka data storage process flow. AI: Artificial Intelligence; AWS: Amazon Web Service; DHIS: District Health Information System; NDoH: Nation Department of Health; S3: Simple Storage Service; SANAC: South African AIDS Council; SFTP: Secure File Transfer Protocol; NoSQL: Not Only Structured Query Language.

Data will be extracted from data sources and put into a staging area before being transformed and loaded into the data repository (Figure 3). We will use the Data Intensive Research Initiative of South Africa (DIRISA) platform, which will enable the provision of much-needed timely information at a level and scale that will improve understanding of HIV heterogeneities. Structured data will be preprocessed and normalized into a standardized format using the Boloka Data Harmonization Tool (Multimedia Appendix 4). The repository will be flexible and updatable for structured data by design [36]. Unstructured data will be stored in the NoSQL data store. Preprocessing will entail data cleaning, transformation, and integration to make the data complete for analysis. Data management will involve assembling multiple big, existing HIV data sources and datasets, capturing, preprocessing, cleaning, integrating, and building them into the Boloka data repository.

For disaggregated data, personal identifiable information will be encrypted and hashed to ensure anonymity and protect privacy. This role is also performed by the appointed honest data broker. Ideally, the Boloka data repository will not store any identifiable personal information, but a procedure in accordance with the POPI Act will be put in place for handling this issue if such information comes with the data. The honest data broker mechanism will be set up to screen the data for compliance in terms of the latter before it passes to the Boloka data repository. The resulting data repository will be responsive to the size and scope of the collected data. It will be dynamic and flexible to accommodate diverse data that has a high number of dimensions.

#### Data Security and Access

The access to the data repository will be secured with login credentials (username and password) in conjunction with multifactor authentication (MFA) to ensure compliance with the POPI Act and avoid any confidentiality breaches as well as contravention of human rights. Access to the repository will be made to the PI and Project Manager through a formal and written request. Login credentials (password) will be updated on a 3-month basis to enhance layers of security and reduce the risk of unauthorized access.

### Stage 5: Translate Data Into Actionable Knowledge

Knowledge created from available data will be applied to address real-world challenges. To ensure that findings are incorporated into program and policy decisions promptly, the study will establish a feedback loop with stakeholders and authorized users throughout the project lifespan. Knowledge sharing will be done through dissemination workshops, consultative meetings, reports, dashboards, data visualization, and other methods deemed appropriate. This will support open communication and 2-way feedback between researchers and stakeholders (Multimedia Appendix 5). This participatory process will contribute towards improved use of available data and narrowing the gap between science and practice. Various forms of analysis will be planned in partnership with stakeholders to answer research questions that are relevant to their respective organizations and national priorities. Ultimately, the translation of research will seek to strengthen health systems and, therefore, improve health outcomes throughout South Africa, specifically for KPs and other vulnerable populations. Our goal is to make the Boloka data repository an accessible tool that can be used by local, regional, and international stakeholders for more effective and efficient health care management, prevention, and programming.

The University of Johannesburg Strategic Communication Department designated official will promote and market the initiative using the University of Johannesburg’s communication technologies, media, and platforms. They will promote public understanding of the study and research outputs throughout. This will include articles in the daily press, magazines, and other popular media, public lectures, and interviews or programs on television or print media.

### Ethical Considerations

Ethics approval (May 2022), renewal (June 2023), and amendment (July 2024) have been secured from the University of Johannesburg, Faculty of Health Sciences, Research Ethics Committee for conducting secondary analysis of data (REC-1504-2022) (Multimedia Appendix 6). Data partners will only share deidentified secondary datasets, and no personal information of the participants will be retrieved as per the POPI Act and the University of Johannesburg DPA, which governs the sharing, storage, and use of personal information. For example, in cases of NDoH, the routine data is aggregated and not at an individual level. The South Africa National HIV Prevalence, Incidence, Behavior and Communication Surveys (SABSSM) data is open access and unique identifiers were used. Data handling, processing, and analysis will be guided by the University of Johannesburg DPA, POPI Act, and data partnership agreement or request form. There is no monetary compensation for the data partners for being part of the Boloka project.

## Results

As of December 2022, the protocol was funded by the South African Medical Research Council following external peer reviews. Subsequently, the study received ethics approval from the University of Johannesburg, Faculty of Health Sciences, Research Ethics Committee. The research assistants, students, and postdoctoral fellows who will partake in the creation of the data repository and subsequent analyses were recruited, onboarded, and received online training and resources in research ethics evaluation (TRREE), management and analysis of data in epidemiology (MADE), data types, and structures. The team will continue to tap into opportunities for professional development training, including sensitivity training for KPs and training on data privacy, specifically regarding the POPI Act and DIRISA functionalities for the duration of the study (January 2024-February 2027). The progress as of September 2024 is described below according to the 5 stages.

### Stage 1: Identify and Engage Key Stakeholders to Develop Meaningful Data Partnerships

#### Overview

The stakeholder engagement is ongoing using the data partner tracking tool (Multimedia Appendix 7) listing potential and current stakeholders and their level of engagement with data (eg, district, provincial, and national) is in place.

#### Research Data

The research team secured open access data from 5 (2002, 2005, 2008, 2012, and 2017) South Africa National HIV Prevalence, Incidence, Behavior and Communication Surveys from the Human Sciences Research Council (HSRC) on January 5, 2023. Since the data is open access, no signing of DPA was required to access and secure the data. Engagement with stakeholders is expected to conclude in December 2026 in terms of finalizing data sharing agreements, and an example of the University of Johannesburg DPA has been shared for consideration by the potential data partners. An engagement with NDoH was done in 2022, and HIV-related routine data was secured (April 2024).

#### Program Data

The research team had a series of meetings with SANAC regarding the Boloka data repository. These meetings have established a strong partnership and system for the coordination of data-sharing partnerships. The partnership with SANAC is essential in ensuring that the Boloka data repository is used to guide national HIV strategy related to KPs. This strong relationship will ensure the complementarity of the Boloka data repository rather than duplication of SANAC’s efforts to develop “The Situation Room,” a central HIV data repository for program data in South Africa. The Situation Room, still in its infancy stages, seeks to enable data checks and balances, dynamic visualization, and sharing of the national, provincial, and district HIV data in the general population to monitor progress toward reaching set targets. The Boloka data repository will be a resource for KP data toward SANAC’s efforts. SANAC introduced the research team to its implementation partners to acquire potential data partners, namely Beyond Zero [37], Networking HIV and AIDS Community of Southern Africa (NACOSA) [38], Sex Worker Education Advocacy Taskforce (SWEAT) [39], Sisonke [40], and AIDS Foundation South Africa (AFSA) [41] as well as Perinatal HIV Research Unit (PHRU) [42].

A series of engagements have been carried out with the respective organizations; the engagements are at different stages. Beyond Zero has been engaged since September 2022, and a final copy of the University of Johannesburg and Beyond Zero data-sharing agreement is under review by the Beyond Zero legal team before it is signed by both parties. A data partnership agreement with PHRU is expected to be finalized by the end of December 2024. For this partnership, the PHRU data-sharing agreement is being developed by their legal department. With AFSA, the agreement is in the early stages of communication. In addition to engagements with SANAC and its implementing partners, the research team has consulted with other community leaders who work or worked with community-based organizations, such as treatment action campaigns.

### Stage 2: Acquire and Collate Heterogeneous Data

#### Overview

Significant progress has been made in securing program data, published research data, and technical reports. The security of the acquired data is prioritized during this interim period; thus, the data have been stored in a secure staging area and will later be moved to appropriate data storage software.

#### Research Data

Open access data from 5 cycles (2002, 2005, 2008, 2012, and 2017) of the population-based multistage cluster cross-sectional of up to 85,000 randomly selected households in South Africa has been secured from the HSRC (January 5, 2023). The South African HIV Behavioural, Sero-status and Media data is within the restricted access level. The data was accessed by completing a non–web-based internet data request form detailing the name of the project, a brief description of the intended use, and the expected date of project completion. The surveys provide data on HIV incidence, prevalence, antiretroviral therapy (ART), viral load suppression, drug resistance, risk behaviors, and HIV care, among others. The Key Population Implementation Science (KPIS) data on HIV testing and engagement in care was secured at Emory University (May 16, 2022) for the degree purpose of postgraduate students. Further, data on HIV testing proficiency was generated by a postgraduate student from the health care facilities in Eastern Cape Province (May 2023).

Data from the Demographic Health Surveys Program has been secured (August 2022). This required registration and submission of a data access request form along with the justification of the request. The demographic health survey collects indicators such as HIV prevalence, prevention and treatment, stigma, and discrimination, as well as sexual behavior. We also secured HIV-related data from the NDoH, and the data includes the following HIV indicators: HIV testing and HIV prevention; ART (ie, ART initiation, ART rate, viral load & CD4 T lymphocyte count testing, ART type, ART adherence); sexually transmitted infections; maternal and neonatal (antenatal HIV test, ART initiation, ART adherence, live birth, infant HIV test); management of inpatients and management of primary health care facility.

There are plans to use data from the 1173 NDoH High Transmission Area sites across South African provinces based in communities that function like clinics and provide services to KPs. The NDoH’s High Transmission Area program reaches key and vulnerable populations with HIV, tuberculosis, and sexually transmitted infections prevention and management services in key hotspots across the country.

#### Program Data

This protocol will also leverage a partnership with the largest HIV service provider for key populations in South Africa, TB HIV Care, a nonprofit organization, building on a decade-long period of dynamic and exciting work; preliminary discussions have already taken place with the Chief Executive Officer. TB HIV Care focuses on preventing, finding, and treating HIV and TB across 7 provinces and 22 districts in South Africa [43]. Its KP program provides services to sex workers and people who use drugs. Furthermore, data-sharing agreements are being drafted and checked for Beyond Zero, NACOSA, and AFSA, as detailed above.

### Stage 3: Assess Data for Accuracy, Relevance, And Quality

#### Overview

The data received in stage 2 went through screening and assessment for accuracy, completeness, and consistency before its placement in the staging area. The data received was screened and filtered for relevance and inclusion by a member of the research team using the FCSM and the Information Quality Assessment Framework [32] where applicable.

#### Research Data

South African HIV Behavioural, Sero-status and Media data has undergone a quality check to assess the completeness of the data received. Using the Needs Assessment Form, the DHIS data received has been checked to assess the relevance of the data to the project aims. The assessment checked that the data included the relevant indicators and if the data was complete. This process has been done by 2 researchers on the team.

### Stage 4: Clean and Store Data in The Boloka Data Repository

The data have been placed in the staging area before being stored in the data repository. South African Medical Research Council/ UJ (SAMRC/UJ) Pan African Centre for Epidemics Research Extramural Unit secured cloud-based storage on the DIRISA platform on June 14, 2024. DIRISA is a “component of the National Integrated Cyberinfrastructure System that coordinates and promotes sound research data management practices and supports data-intensive research” [44]. The access will grant the University of Johannesburg permission to use the DIRISA platform, along with access to the support tools and resources. The resources have been shared to provide access to DIRISA training materials. Through this platform, KP data will be harmonized into a centralized storage area that is managed and protected. All data is to be cleaned and converted into a standardized format to create a structured, flexible, and updatable data repository. The designing of the Boloka data repository will be completed by December 2026. Multimedia Appendix 8 shows the summary of planned and executed milestones for this project.

### Stage 5: Translate Data Into Actionable Knowledge

Authorized users and stakeholders will have the capability to generate customized reports and export the data to applications such as Stata software (StataCorp) for further dissemination. Authorized users and stakeholders will submit a data request before receiving log-in credentials to access the data repository. Initial secondary data analyses using analytic methods attuned to the structure of available data, including cross-sectional and longitudinal analyses, are being conducted to improve our understanding of HIV among KPs for a targeted response. The proposed analyses will be a transdisciplinary collaborative effort to enable joint application of theories and methods to share conceptual frameworks, innovations, and best practices for solving public health problems. In this regard, the proposed analyses will be jointly finalized with program partners to identify priority research questions that will measure program progress, guide programmatic decision-making, and ultimately improve the response to HIV.

Data synthesis and initial analyses are being planned with a transdisciplinary team of infectious disease epidemiologists, public health scientists, statisticians, data scientists, data analysts, implementing partners, and data partners who will bring complementary expertise on global key populations insights, key population modeling, epidemiology, human rights, and the development of a sustainable data platform. We have already worked with some of the data partners to determine the research agenda that targets policy and practice outcomes, that is, determining common research questions, models, and methods to improve South Africa’s HIV response. The doctoral students who will use the Boloka data repository for their dissertations have begun writing their research proposals and seeking ethical approval.

**Figure 4:**
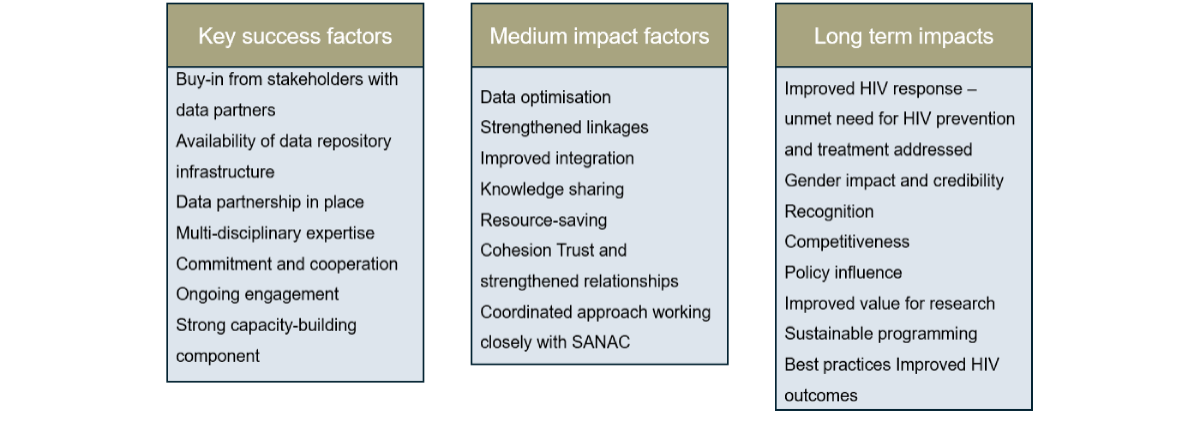
Key success factors for Boloka data repository and envisaged impacts. SANAC: South African National AIDS Council.

Some factors are critical for the success of this project in achieving the envisaged medium- and long-term impacts, as shown in Figure 4.

## Discussion

### Principal Findings

It is anticipated that there will be data challenges that are common across projects of this nature due to the POPI Act. The University of Johannesburg has already established a mechanism to address these. Potential POPI Act–related challenges have been highlighted in the University of Johannesburg DPA, which is standard for all data-driven projects.

The Boloka data repository will be a lasting resource for the country to guide regional policies and strategies to address HIV, particularly among KPs. Therefore, in addition to traditional research dissemination led by the research team and the proposed feedback loop (Multimedia Appendix 4), the data repository will be widely accessible to all data partners and stakeholders. This co-ownership with stakeholders will enable alternative possibilities for analyses and translation of data to real-world settings. The knowledge, historical context, and applied understanding that stakeholders possess provide opportunities for transformative change. The protocol will provide the opportunity to document lessons learned into a knowledge base, which may be applied to other countries in Sub-Saharan Africa.

The Boloka data repository will contribute to improved storage, retrieval, and access to KP data; linkages of surveillance data collection systems and other data collection efforts; integrated data reporting for monitoring national trends and patterns; recommendations for research, policy, and planning; as well as changes in current and future research. The Boloka data repository will provide a richer empirical basis for policy and program debates in a relatively neglected area of health research in the country. The data, along with its analyses and research outcomes, can form the basis of regularly reported statistics by stakeholders. The Boloka data repository will be maintained up to date to serve as a real-time sustainable resource to guide HIV planning, resource allocation, policy formulation, and programming.

### Strengths and Limitations

The Boloka project seeks to build a complex data repository to house and analyze big heterogeneous data from KPs to guide the HIV response in South Africa and, ultimately, SSA. This initiative is crucial in developing policy recommendations for innovative methods towards ending the HIV epidemic in 2030. The Boloka initiative is a novel approach that has the potential to enhance knowledge translation. It will also provide opportunities for capacity-building among academic trainees and stakeholders. Furthermore, the Boloka repository will contribute to the optimization of the HIV data systems necessary for epidemic control. Various data types will be used through integrated analysis to uncover latent outcomes. However, the use of secondary data has limitations as no changes can be made toward improving internal validity. The data requested are from cross-sectional surveys that lack the ability to establish causality. Mitigation of data security, integrity, and confidentiality issues has been described in the relevant sections.

### Conclusions

We posit that a truly “complete” data infrastructure that systematically and rigorously integrates empirical, contextual, observational, and programmatic data for KPs will not only improve our understanding of local epidemics but will also improve HIV interventions and policies. Furthermore, it will inform future research directions and become an incredible institutional mechanism for epidemiological and public health training. Achieving epidemic control in South Africa necessitates moving beyond HIV data silos, harnessing underused heterogeneous data, and conducting unconventional analyses. Creating a comprehensive and accessible data infrastructure inclusive of heterogeneous data will improve our understanding of HIV among KPs and accelerate the production of high-quality evidence. Empowered with this evidence, scientists, program leaders, community stakeholders, and policy makers will be able to tailor and optimize HIV service delivery to the most vulnerable populations, thus making the South African National Strategic Plan goal possible. The amassed evidence will provide opportunities for comprehensive and innovative analyses that seek to address priority research questions to improve our understanding of HIV among KPs, assist in setting program targets, guide programmatic decision-making, and ultimately improve the response to HIV in South Africa and other Sub-Saharan African countries. Efforts will be made to incorporate the findings from this study into the South African HIV response strategy.

## Appendix

Multimedia Appendix 1 List of potential and current data partners as well as nature of data.

Multimedia Appendix 2 Boloka Data Indicator Tool.

Multimedia Appendix 3 Global HIV Quality Assessment Tool.

Multimedia Appendix 4 Boloka Harmonization Tool.

Multimedia Appendix 5 Proposed feedback loop.

Multimedia Appendix 6 Ethics approval letter.

Multimedia Appendix 7 Data Partners Tracking Tool.

Multimedia Appendix 8 Summary of planned and executed milestones for this project.

## Abbreviations

AFSA: AIDS Foundation South Africa
ART: antiretroviral therapy
DHIS: District Health Information System
DIRISA: Data Intensive Research Initiative of South Africa
DPA: Data Processing Agreement
DUA: Data User Agreement
FCSM: Federal Committee on Statistical Methodology
HSRC: Human Sciences Research Council
KP: key population
KPIS: Key Population Implementation Science
MADE: management and analysis of data in epidemiology
MFA: multifactor authentication
NACOSA: Networking HIV and AIDS Community of Southern Africa
NDoH: National Department of Health
PACER: Pan African Centre for Epidemics Research
PHRU: Perinatal HIV Research Unit
PI: Principal Investigator
POPI: Protection of Personal Information
SABSSM: South Africa National HIV Prevalence, Incidence, Behavior and Communication Surveys
SAMRC: South African Medical Research Council
SANAC: South African National AIDS Council
SWEAT: Sex Worker Education Advocacy Taskforce
tPAFt: transmission population attributable fraction over time
TRREE: training and resources in research ethics evaluation
