# Supplementary material for: Harnessing Big Heterogeneous Data to Evaluate the Potential Impact of HIV Responses Among Key Populations in Sub-Saharan Africa: Protocol for the Boloka Data Repository Initiative
Source: JMIR Res Protoc. 2025 Jan 22;14:e63583. doi: 10.2196/63583 (PMC11799808; doi:10.2196/63583)
Supplement: Multimedia Appendix 1 [file resprot_v14i1e63583_app1.docx]

**Multimedia Appendix 1: List of potential and current data partners as well as nature of data**

| **Data Type** | **Data Source** | **Purpose of Data Collection** | **Example(s)** |
| --- | --- | --- | --- |
| Survey Data | Science Councils, e.g., Human Sciences Research Council, South African Medical Research Council, Government Departments, e.g., National Department of Health, Social Development, Basic Education, etc. | Data to monitor HIV indicators | Country-specific surveys, e.g., data from bio behavioural surveillance surveys among FSW and MSM, HIV prevalence survey amongst transgender women (TGW) in South Africa, South African National HIV, Prevalence, Incidence, Behaviour and Communication surveys (SABSSM surveys), South African Men’s Health Monitoring Study (SAMHMS), HSRC Botshelo Ba Trans, South African National Health and Nutrition Examination Survey (SANHANES), HIV Surveys, Sexual and reproductive health surveys, periodic South African Stigma Index surveys, the Governance, Public Safety and Justice survey (GPSJS), Demographic Health surveys, online HIV surveys, household surveys, Demographic Health Surveys |
| Research Data | Academic institutions, research institutions, collaborators | Research conducted to answer priority HIV questions for the country -observational, implementation science, and experimental studies | Data from collaborative studies with Johns Hopkins University, Emory University, Centres for Disease Control and Prevention, Health Science Research Council, Desmond Tutu HIV Foundation, and University of California San Francisco |
| Program Data from implementing partners | Implementing Partners and main funders (NGOs, community- based organizations (CBOs), Global Fund, PEPFAR) | Routinely collected data at facility, district level and countrywide captured by partners for government reporting population size estimates (PSEs) for FSW and MSM | AIDS Foundation of South Africa (AFSA), Networking HIV and AIDS Community of Southern Africa (NACOSA, TB/HIV Care; Beyond Zero, Aurum, South African Network of People who Use Drugs (SANPUD) |
| Program data from national government departments | Government departments, e.g., Department of Health, Social Development and Basic Education | Routinely collected patient-level data and reporting purposes | Electronic health information management system, District Health Management system (DHIS), Tier.net, High Transmission Area data |
| Data from modeling studies | University of Cape Town | Estimates on key HIV-related indicators for guiding programming and policies | Data from Thembisa and Naomi models |
| Program data from community-led programs | Community-led monitoring system, developed by organizations representing people living with HIV | Routinely collected data collected for community level monitoring | Ritshidze, High Transmission Area Program |
| Reports | SANAC Report, Country and departmental annual reports, and organizational reports | Collate information from different sources to gauge the response | Data from the Annual Global AIDS Monitoring Report, NSP Mid-term and Annual Reports, Quarterly Factsheets on NSP, UNAIDS reports, NAC reports, WHO Reports, UNICEF reports |
