# Supplementary material for: Harnessing Big Heterogeneous Data to Evaluate the Potential Impact of HIV Responses Among Key Populations in Sub-Saharan Africa: Protocol for the Boloka Data Repository Initiative
Source: JMIR Res Protoc. 2025 Jan 22;14:e63583. doi: 10.2196/63583 (PMC11799808; doi:10.2196/63583)
Supplement: Multimedia Appendix 2 [file resprot_v14i1e63583_app2.docx]

**Multimedia Appendix 2: Boloka Data Indicator Tool**
