# Supplementary figures and images for: Harnessing Big Heterogeneous Data to Evaluate the Potential Impact of HIV Responses Among Key Populations in Sub-Saharan Africa: Protocol for the Boloka Data Repository Initiative
Source: JMIR Res Protoc. 2025 Jan 22;14:e63583. doi: 10.2196/63583 (PMC11799808; doi:10.2196/63583)

**Multimedia Appendix 3: Global HIV Quality Assessment Tool**

**
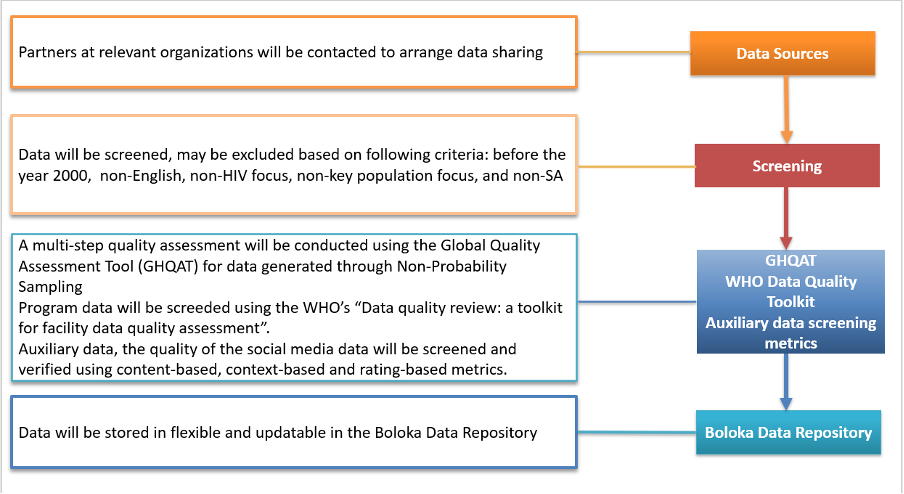
**

Supplement: Multimedia Appendix 3 [file resprot_v14i1e63583_app3.docx]

**Multimedia Appendix 4: Boloka Harmonisation Tool**


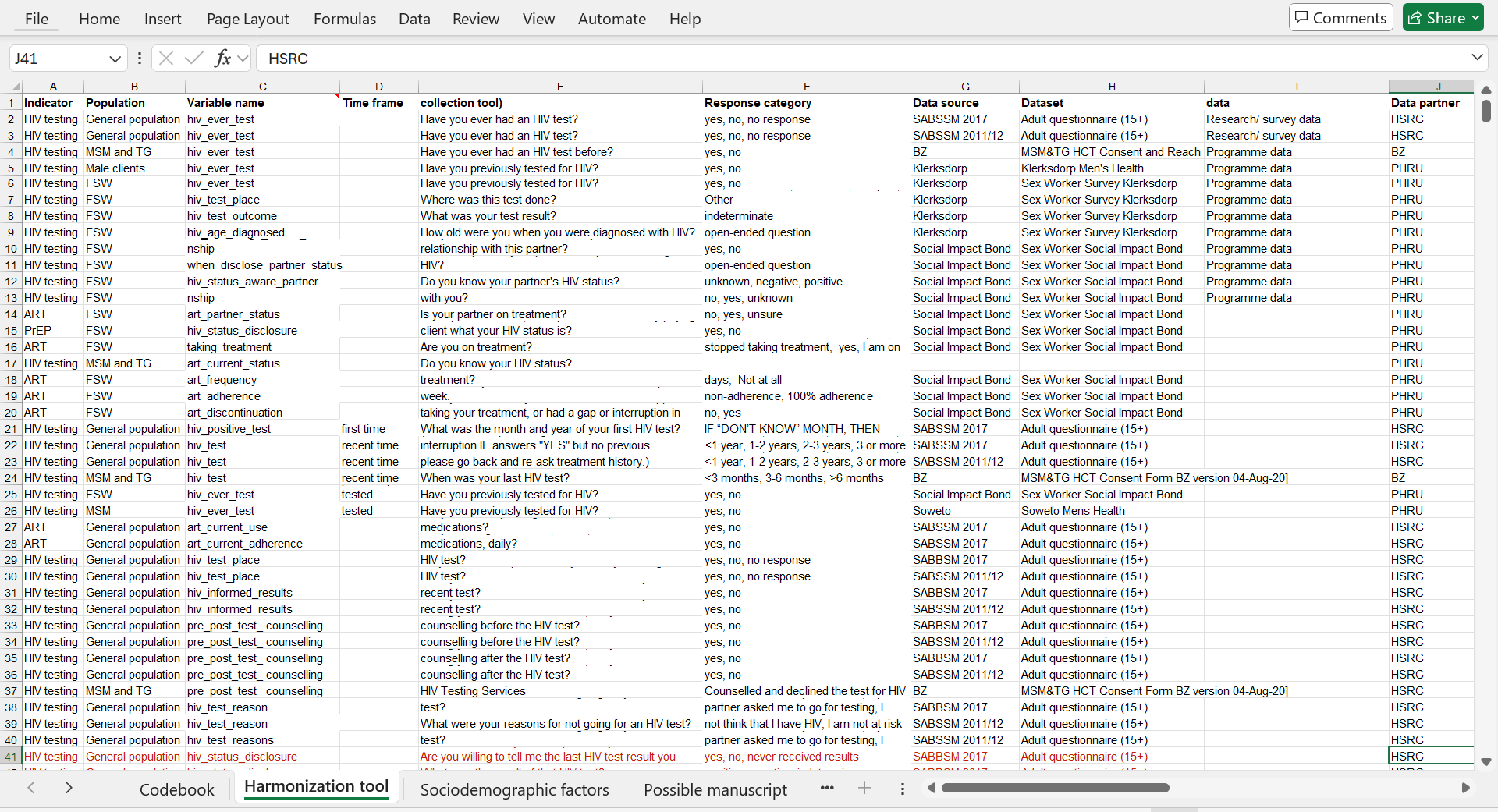

Supplement: Multimedia Appendix 4 [file resprot_v14i1e63583_app4.docx]

**Multimedia Appendix 5: Proposed feedback loop**


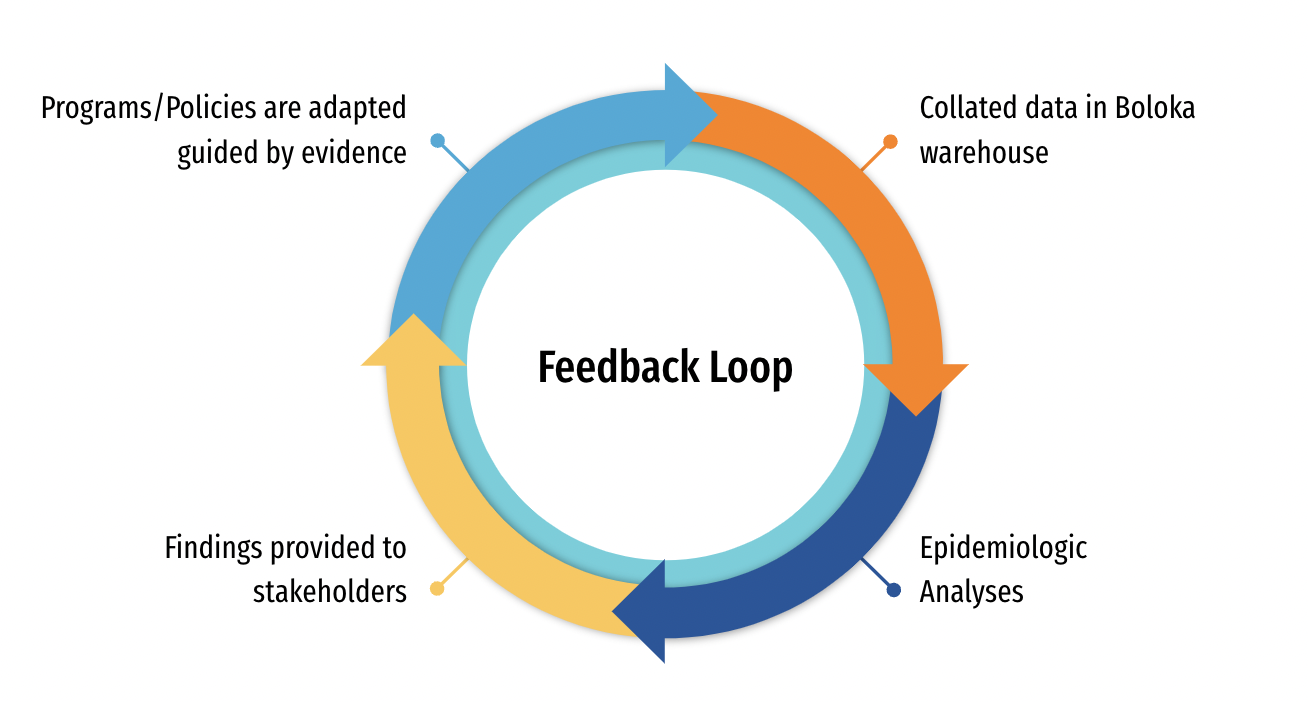

Supplement: Multimedia Appendix 5 [file resprot_v14i1e63583_app5.docx]

**Multimedia Appendix 6: Ethics approval letter**


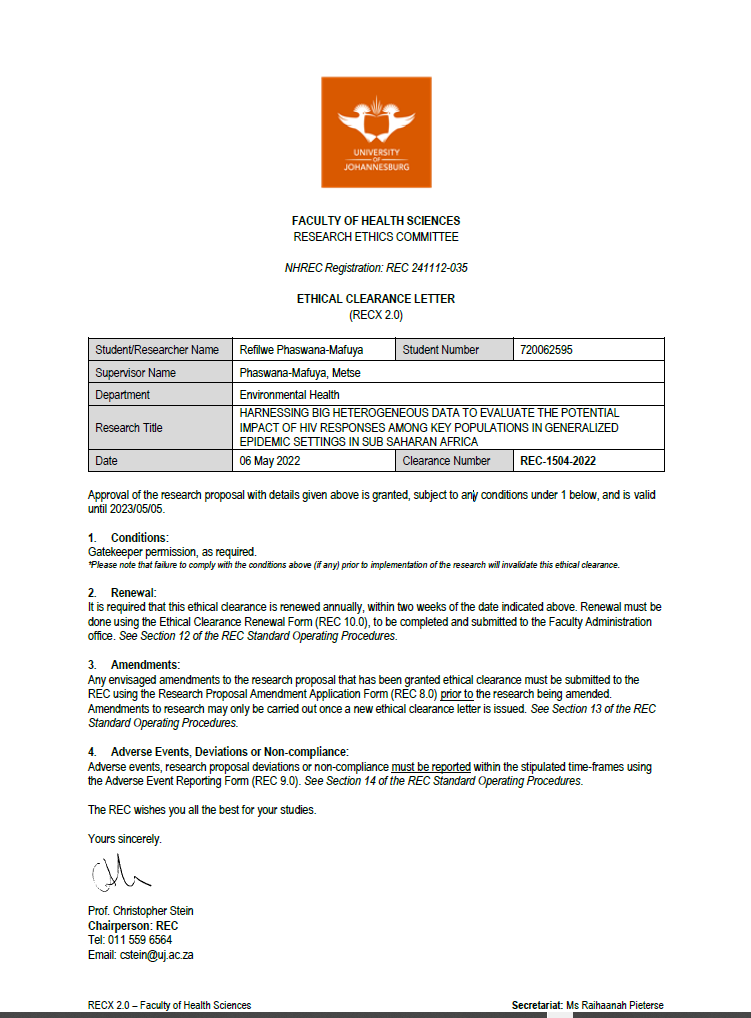

Supplement: Multimedia Appendix 6 [file resprot_v14i1e63583_app6.docx]

**Multimedia Appendix 7: Data Partners Tracking Tool**


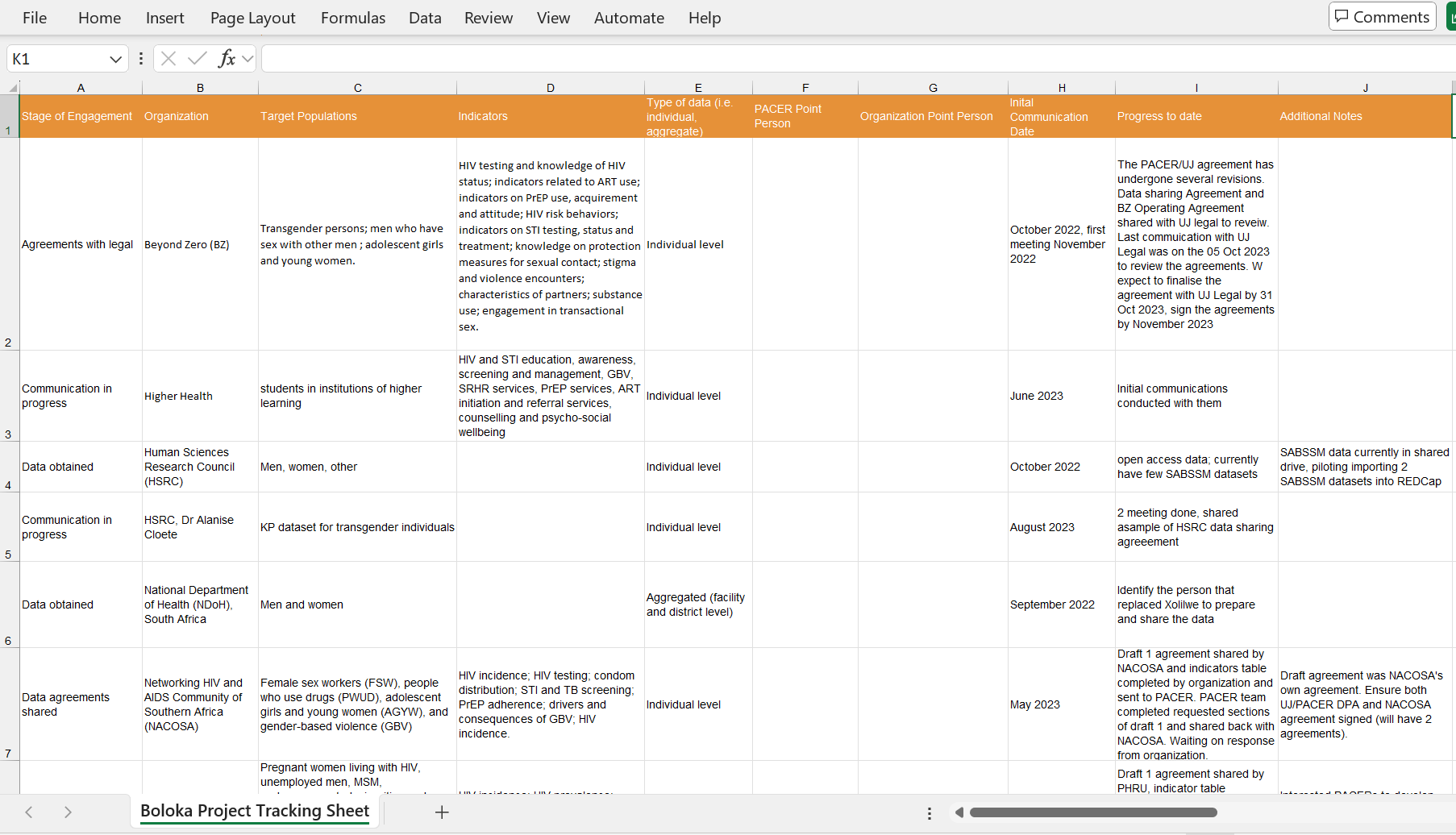

Supplement: Multimedia Appendix 7 [file resprot_v14i1e63583_app7.docx]
