# Supplementary material for: Harnessing Big Heterogeneous Data to Evaluate the Potential Impact of HIV Responses Among Key Populations in Sub-Saharan Africa: Protocol for the Boloka Data Repository Initiative
Source: JMIR Res Protoc. 2025 Jan 22;14:e63583. doi: 10.2196/63583 (PMC11799808; doi:10.2196/63583)
Supplement: Multimedia Appendix 8 [file resprot_v14i1e63583_app8.docx]

**Multimedia Appendix 8: Summary of planned and executed milestones for this project**

| **Activity** | **2024** | **2025** | **2026** | **2027** |
| --- | --- | --- | --- | --- |
| **Study approvals** | | | | |
| Yearly ethics renewal |  |  |  |  |
| **Study trainings** | | | | |
| Yearly refresher study training with all the project personnel i.e., ethics, study procedures, POPI Act training |  |  |  |  |
| **Acquire and collate heterogeneous data** | | | | |
| Assess and develop meaningful data partnerships and collaborations; data sharing agreements |  |  |  |  |
| Obtaining & extracting data from multiple data sources |  |  |  |  |
| **Assess data for accuracy, relevance, and quality** | | | | |
| Pre-screening for relevance and inclusion, Capturing, pre-processing data, sorting, and storing data |  |  |  |  |
| Synthesize/collate/merging data from various sources, critical review according to the checklist, quality control |  |  |  |  |
| **Clean and store data in the data repository** | | | | |
| Clean and filter the data |  |  |  |  |
| Store the data on DIRISA platform or similar |  |  |  |  |
| Set up the Boloka Data repository |  |  |  |  |
| **Translate data into actionable knowledge** | | | | |
| Data integration and data flow control, maintenance, updates |  |  |  |  |
| License fees, adoption, implementation, analysis, and visualization of data |  |  |  |  |
| The Boloka Project Protocol Paper – Submission for publication |  |  |  |  |
